# Adolescents’ spontaneous recall of food and beverage advertisements on digital media: a cross-sectional survey in Montevideo, Uruguay

**DOI:** 10.1017/S1368980025101481

**Published:** 2025-12-03

**Authors:** Gastón Ares, Leandro Machín, Lucía Antúnez, Florencia Alcaire, Virginia Natero, Vanessa Gugliucci, Carolina de León, Tobias Otterbring

**Affiliations:** 1 Sensometrics & Consumer Science, Instituto Polo Tecnológico de Pando, Facultad de Química, Universidad de la República, By Pass de Rutas 8 y 101 s/n, CP 91000, Pando, Uruguay; 2 Centro de Investigación Básica en Psicología, Facultad de Psicología, Universidad de la República, Tristán Narvaja 1674, CP 11200, Montevideo, Uruguay; 3 Escuela de Nutrición, Universidad de la República, Montevideo, Uruguay; 4 División Salud, Intendencia de Montevideo, Montevideo, Uruguay; 5 School of Business and Law, Department of Management, University of Agder, Universitetsveien 17, 4630 Kristiansand, Norway

**Keywords:** Marketing, Digital marketing, Social media, Food environment, Adolescence

## Abstract

**Objective:**

Explore adolescents’ recall of food and beverage advertisements in digital media, while evaluating associations between socio-demographic characteristics and advertisement recall.

**Design:**

Recruitment took place using a two-stage cluster probability-based sampling approach. Thirty-nine high schools stratified by type (public *v*. private) were included, with one class within each grade randomly selected, wherein attending students (*n* 1542; age range: 11–19 years) received a paper-and-pencil questionnaire for completion in their homes. Participants indicated their spontaneous recall of food and beverage advertisements on social media and provided socio-demographic information. Individual responses to an open-ended question were graphically represented using a world cloud, after which the data were analysed through content analysis based on inductive coding.

**Setting:**

The study was conducted in Montevideo, the capital city of Uruguay, which is a high-income South American country with a high prevalence of overweight and obesity among adolescents.

**Participants:**

A total of 1542 adolescents attending public and private high schools participated.

**Results:**

Almost nine of ten adolescents (87·6 %) reported having seen a food or beverage advertisement on digital media and more than three of four (76·1 %) could spontaneously recall at least one such advertisement. The three most frequently used words for spontaneous recall were ‘McDonalds’, ‘Coke’ and ‘burgers’, whereas the three most frequently mentioned product categories were ‘Fast-food and fast-food restaurants’, ‘soft drinks’ and ‘savoury snacks’. Some socio-demographic differences emerged.

**Conclusions:**

The findings stress the need to implement mandatory regulatory approaches to reduce adolescent exposure to digital marketing of unhealthy foods and beverages.

Adolescents’ exposure to digital food marketing has received increasing attention in recent years given the potential deleterious effects of such exposure on nutrition and health outcomes^(1,2)^. Adolescents are avid users of digital media and are particularly susceptible to the persuasive effects of unhealthy food marketing due to a series of neurodevelopmental and social mechanisms that reduce inhibitory control, heighten sensitivity to rewards and increase vulnerability to social pressure^(3–5)^.

Through a hierarchy of effects, exposure to digital marketing can encourage consumption of foods high in nutrients associated with non-communicable diseases, which may contribute to the development of overweight, obesity and non-communicable diseases^(6,7)^. In this sense, some observational studies have shown that exposure to digital marketing of unhealthy foods and beverages is associated with increased consumption of such products^(8–12)^.These results, together with the increasing relevance of digital marketing as part of the promotional activities of food companies^(13–15)^, underscore the need for additional empirical evidence about the nature, extent and impact of digital marketing of unhealthy foods across the globe^(1,16)^.

The effectiveness of an advertisement largely depends on its ability to capture attention^(6,17)^. If this occurs, advertisements can be stored in long-term memory and influence purchase decisions^(18,19)^. Although certain marketing and advertising practices can exert an influence on consumers’ purchase behaviour and product preferences even without conscious awareness through phenomena such as priming and peripheral cues, spontaneously recalled or unprompted advertisements can be regarded as those with the highest ability to influence behaviour at the point of purchase, particularly when making impulsive decisions^(18,20,21)^. This may be relevant for products eliciting hedonic and emotional reactions, such as ultra-processed products, which are vastly over-represented among those that consumers purchase on impulse^(22–24)^.

Exposure to and recall of digital food marketing may vary with socio-demographic characteristics due to the targeting strategies and differences in food habits and preferences. However, there is limited knowledge about the existence of socio-demographic differences in exposure to digital food marketing in adolescence, although a recent study showed differences in the content of food and beverage advertisements in digital media boys and girls were exposed to^(25)^.

While advertising influences individuals through multiple pathways, the present work aimed at exploring adolescents’ spontaneous recall of food and beverage advertisements in digital media. As a secondary objective, the study evaluated associations between socio-demographic characteristics and advertisement recall. The study was conducted in Montevideo, the capital city of Uruguay, a high-income South American country with one of highest prevalence of overweight and obesity among adolescents in the region (33·6 %)^(26)^. At the time of the study, Uruguay had not implemented any regulations or guidelines on food marketing on mass media, including digital food marketing.

## Methods

The present study relies on data from a previously published cross-sectional observational study on Uruguayan adolescents’ exposure to digital food marketing^(12)^. A survey was used to analyse self-reported exposure to digital food marketing among Uruguayan adolescents and its association with food consumption frequency. The present study focuses on spontaneous data on recall of food and beverages advertisements, assessed through open-ended question, which has not been analysed in the previously published paper.

### Participants

A total of 1542 adolescents attending public and private high schools in Montevideo participated in the study. They were recruited using a two-stage cluster probability-based sampling approach. First, a sample of thirty-nine high schools, stratified by type (public *v*. private), was obtained. All the selected high schools agreed to participate in the study. One class within each grade was randomly selected in each high school, and all students received a questionnaire. Students not attending school the day the questionnaires were distributed were excluded. The number of participants (*n* 1542) corresponded to 38·8 % of the total number of students in the selected classes (*n* 3974).

Participants’ age ranged from 11 to 19 years (Mean = 14·3, sd = 1·8), 56·2 % were females, and the mean total daily digital media use was 5·0 h (sd = 3·2). Participants’ socio-demographic characteristics are summarised in online supplementary material, Supplemental Table 1.

### Questionnaire

The questionnaire included four main sections: (i) exposure to advertisements on digital food marketing, (ii) food consumption frequency, (iii) social media use and (iv) socio-demographic questions. The present study focuses on the second question of the first section, which asked participants to indicate their spontaneous recall of food and beverage advertisements on social media. Participants were first asked to indicate their frequency of exposure to digital food marketing (*Have you seen advertisements of foods and beverages on social media or websites in the last week?*) using the following response options: ‘Yes, more than once a day’, ‘Yes, once a day’, ‘Yes, several times a week’, ‘Yes, once a week’ and ‘No, I haven’t seen any advertisement’. Then, they were asked to answer the following open-ended question to assess their spontaneous recall of food and beverage advertisements: *What advertisements of food and beverages do you recall seeing on social media*?

The present study also uses self-reported social media use and socio-demographic data from the last two sections of the questionnaire. Participants were asked to indicate how much time they normally used different digital media on a weekday using the following response options: ‘I don’t use’, ‘Less than 15 min’, ‘15 to 30 min’, ‘30 min to 1 h’, ‘1 to 2 h’, ‘2 to 3 h’, ‘3 to 4 h’ and ‘More than 4 h’. Responses were recoded using the midpoint of the range (e.g. 15 to 30 min was recoded to 22·5 min). The ‘More than 4 h’ option was recoded as 240 min (4 h). Participants’ total exposure to digital media (in hours) was calculated by summing the reported usage time across eight platforms: Instagram, TikTok, Facebook, Twitter, YouTube, Snapchat, Twitch and Website browsing.

Participants also indicated their gender (male, female and other), age and neighbourhood of residence. Socio-economic status was estimated using the score of the neighbourhood in the national socio-economic status index, which ranges from 0 to 14^(27)^. Socio-economic status was defined as follows: < 4 low socio-economic status, ≥ 4 and < 11 medium socio-economic status and ≥ 11 high socio-economic status.

### Data collection

Paper-based questionnaires were distributed by high school staff, and participants completed it at their own homes. For adolescents under 18, written informed consent was required from a parent or guardian, along with written assent from the adolescent. For those aged 18 or older, only the adolescent’s informed consent was required. No compensation for participation was provided. Participants returned the completed questionnaires to high school staff, who contacted the research team to pick them up. Data collection was performed between April and July 2024.

### Data analyses

Individual responses to the open-ended question were graphically represented using a world cloud after removing stop-words. Then, the data were analysed using content analysis based on inductive coding^(28)^. One of the researchers developed an initial coding frame to group individual responses into categories. The coding was revised by another researcher, who suggested minor changes. Both researchers independently coded all the data using the agreed coding frame. The categories included in the coding frame referred to specific products, brands, categories of foods and beverages and/or to specific characteristics of foods and beverages, as described in online supplementary material, Supplemental Table 2. Binary variables were created to indicate whether each participant mentioned responses related to each of the categories or not (1/0, respectively). The agreement between the two researchers was excellent, as indicated by Cohen’s kappa (κ > 0·96 for all categories)^(29)^. The number and percentage of participants providing responses related to each of the categories were calculated. Overarching codes were created to group the categories (see online supplementary material, Supplemental Table 2).

Logistic regressions in R^(30)^ were used to analyse associations between recall of exposure to advertisements of each of the identified categories and socio-demographic variables. The binary variables indicating advertisement recall were used as the dependent variable, whereas the following individual characteristics were considered as independent variables: gender (male *v*. female), age (range 11–14 years old *v*. 15–19 years old), socio-economic status (low *v*. high and low *v*. medium) and digital media use (in hours). Separate regressions were run for each of the categories mentioned by at least 2 % of the participants. Participants with missing data (*n* 24) or whose self-reported gender identities (*n* 9) different from males or females were not considered in the analyses.

## Results

The percentage of participants who reported having seen a food or beverage advertisement on digital media in the week prior to the survey was 87·6 %. The majority of these participants (86·8 %, *n* 1173) was able to spontaneously recall at least one advertisement. The remaining 13·2 % (*n* 178) did not answer the open-ended question or indicated that they were not able to recall any specific food and beverage advertisement (e.g. ‘I don’t remember exactly but I know I’ve seen a lot;’ ‘I can’t remember a specific advertisement’). Thus, 76·1 % of the participants were able to recall at least one food and beverage advertisement they had seen in digital media.

**Figure 1.**
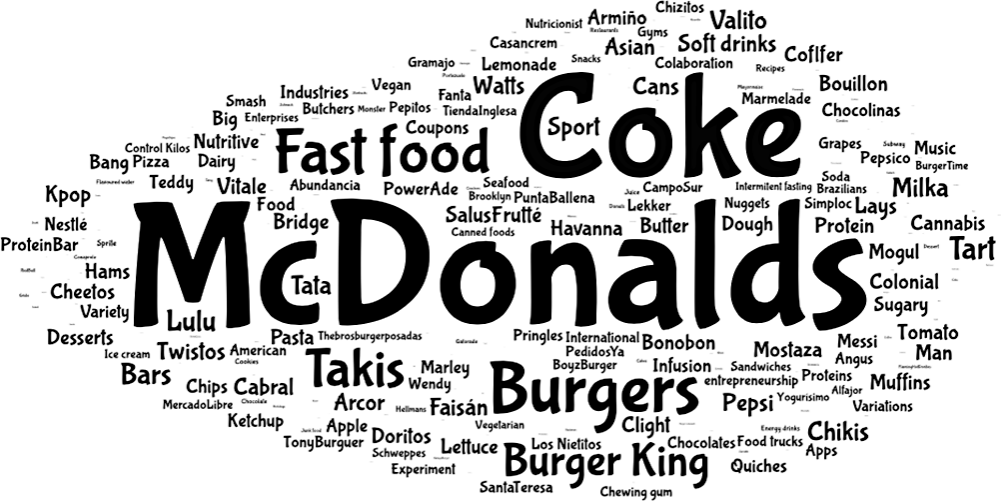
Word cloud summarizing results from the open-ended question on spontaneous recall of food and beverage advertisements in digital media in the week prior to the survey.

When asked about their spontaneous recall of food and beverage advertisements on digital media, participants provided references to specific brands, products, product categories, food characteristics and/or characteristics of the advertisements, as shown in Figure 1. The six most frequently used words were ‘McDonalds’ (*n* 475), ‘Coke’ (*n* 399), ‘burgers’ (*n* 195), ‘fast-food’ (*n* 172), ‘Takis’ [brand of savoury snacks] (*n* 146) and ‘Burger King’ (*n* 141). The rest of the words were mentioned by less than 100 participants.

The most frequently recalled advertisements corresponded to fast-food and ultra-processed products (Table 1). Specifically, three product categories emerged as the most frequently mentioned: ‘Fast-food and fast-food restaurants’ (51·6 %, *n* 795), ‘soft drinks’ (*n* 510, 33·1 %) and ‘savoury snacks’ (*n* 263, 17·1 %). A wide range of other product categories were mentioned, but their frequency was lower than 6 %. Very few participants recalled seeing advertisements of culinary preparations and fruits and vegetables (Table 1).

**Table 1.** Number and percentage of participants who spontaneously recalled advertisements related to each of the categories identified in the content analysis and results of the logistic regression analysing the association between socio-demographic characteristics and advertisement recall (expressed as odds ratios with 95 % confidence intervals)

Category	Number of participants	Percentage of participants (%)	Odds-ratio								Social media use (expressed in hours)	
			Gender		Age range		Socio-economic status					
			Male		15–19 years old		Medium		High			
			OR	95 % CI	OR	95 % CI	OR	95 % CI	OR	95 % CI	OR	95 % CI
Outlets selling prepared foods												
Fast-food and fast-food restaurants	795	51·6	0·95	0·90, 1·00	**1·06**	**1·01, 1·12**	**1·15**	**1·06, 1·23**	**1·18**	**1·09, 1·28**	1·01	1·00, 1·02
Food delivery apps	43	2·8	1·00	0·99, 1·02	1·01	0·99, 1·03	0·99	0·96, 1·02	1·00	0·97, 1·02	1·00	1·00, 1·00
Restaurants (generic)	20	1·3	–		–		–		–		–	
Sushi restaurants	11	0·7	–		–		–		–		–	
Vegan foods and restaurants	1	0·1	–		–		–		–		–	
Ultra-processed products												
Soft drinks	510	33·1	1·02	0·97, 1·07	**1·06**	**1·01, 1·11**	1·02	0·95, 1·09	0·99	0·91, 1·07	1·00	0·99, 1·01
Savoury snacks	263	17·1	0·98	0·94, 1·02	1·04	0·99, 1·07	1·04	0·97, 1·10	**1·09**	**1·02, 1·16**	1·00	0·99, 1·01
Energy drinks	84	5·4	1·01	0·99, 1·04	1·01	0·98, 1·03	1·00	0·97, 1·03	1·01	0·97, 1·05	1·00	1·00, 1·00
Flavoured water	71	4·6	0·99	0·97, 1·02	0·98	0·96, 1·01	1·00	0·97, 1·03	1·01	0·98, 1·05	1·00	1·00, 1·00
Chocolates and confectionary	54	3·5	0·97	0·96, 1·02	1·00	0·98, 1·02	1·00	0·97, 1·09	0·99	0·96, 1·02	1·00	1·00, 1·00
Condiments (e.g. ketchup, mayonnaise)	53	3·4	1·00	0·98, 1·02	1·00	0·98, 1·02	1·02	0·99, 1·05	1·00	0·97, 1·04	1·00	1·00, 1·00
Cookies and crackers	47	3·0	**0·96**	**0·95, 0·98**	1·01	1·00, 1·03	1·02	0·99, 1·05	**1·04**	**1·01, 1·07**	1·00	0·99, 1·00
Ice-creams	46	3·0	0·99	0·97, 1·00	1·00	0·98, 1·01	1·02	0·99, 1·04	1·03	1·00, 1·05	1·00	1·00, 1·01
Alfajores	46	3·0	1·01	0·99, 1·03	1·00	0·98, 1·02	1·02	1·00, 1·05	1·03	1·00, 1·06	1·00	1·00, 1·00
Cold cuts and sausages	43	2·8	1·01	0·99, 1·02	1·01	0·99, 1·02	1·02	0·99, 1·04	1·01	0·99, 1·04	1·00	1·00, 1·00
Bakery products	33	2·1	0·99	0·98, 1·01	1·01	1·00, 1·03	1·00	0·98, 1·02	1·00	0·07, 1·02	1·00	1·00, 1·00
Powdered drinks and bottled juices	27	1·8	–		–		–		–		–	
Dairy products (e.g. yogurt, milk desserts)	21	1·4	–		–		–		–		–	
Instant soups and bouillon cubes	9	0·6	–		–		–		–		–	
Breakfast cereals and cereal bars	9	0·6	–		–		–		–		–	
Marmalade and dulce de leche	4	0·3	–		–		–		–		–	
Natural foods and culinary preparations												
Recipes	42	2·7	**0·98**	**0·96, 0·99**	0·99	0·98, 1·01	1·01	0·99, 1·03	1·01	0·98, 1·03	1·00	1·00, 1·00
Healthy foods	32	2·1	0·99	0·98, 1·01	1·01	0·99, 1·02	1·01	0·99, 1·03	0·99	0·97, 1·01	1·00	1·00, 1·00
Fruits and vegetables	8	0·5	–		–		–		–		–	
Others												
Desserts	29	1·9	–		–		–		–		–	
Alcoholic beverages	16	1·0	–		–		–		–		–	
Promotions (e.g. discounts)	11	0·7	–		–		–		–		–	
Coffee	5	0·3	–		–		–		–		–	
Weight loss	3	0·2	–		–		–		–		–	
Yerba mate	2	0·1	–		–		–		–		–	

The references in the model were female for gender, 11–14 years for age and low for socio-economic status. Odds ratios highlighted in bold are statistically significant at the conventional level of statistical significance (*P* < 0·05).

Significant associations between socio-demographic characteristics and recall of advertisements were found for four of the thirty categories (Table 1). Males were less likely than females to recall advertisements of cookies and crackers or recipes, whereas participants aged 15–19 years were 6 % more likely to recall advertisements of soft drinks and fast-food and fast-food restaurants than those aged 11–14 years. Finally, participants from high socio-economic status were more likely to spontaneously recall having seen advertisements of three categories than those from low socio-economic status: fast-food and fast-food restaurants, savoury snacks and cookies and crackers. For fast food and fast-food restaurants, the same difference was observed between participants from medium and low socio-economic status.

## Discussion

Results from the present work contribute to the increasing body of literature showing that adolescents are frequently exposed to food and beverage advertising in digital media^(8,31)^. Participants mostly recalled seeing advertisements of fast-food and ultra-processed products, whereas references to the foods recommended by the Uruguayan dietary guidelines (e.g. unprocessed foods and culinary preparations)^(32)^ were scarce. This result is aligned with a previous qualitative study exploring the experiences of Uruguayan adolescents with digital food marketing^(33)^.

Advertisements of fast-food, soft drinks and savory snacks were the most frequently spontaneously recalled by participants. These categories have been reported to be frequently promoted on Instagram in the country^(34–36)^ and have been identified as the most prevalent in studies assessing adolescent exposure to digital food marketing^(25,31,37–41)^. Exposure to digital marketing of these categories encourages adolescents to consume them^(8–12)^. In this sense, participants frequently mentioned specific brands and products, which has the potential to influence behaviour at the point of purchase^(18,20,21)^.

Results from the present work show socio-demographic differences in digital food marketing recall. Gender, age and socio-economic status were significantly associated with the likelihood of recalling advertisements of five categories. This can be explained by the targeting algorithms of social media^(16)^ as well as by differences in attention to the advertisements as a consequence of differences in habits and preferences^(42)^. Gender differences in marketing exposure have been previously reported by other authors in the context of different media^(43)^, including digital media^(25)^. To the best of the authors’ knowledge, this is the first study to examine differences in spontaneous recall of digital food marketing based on adolescents’ socio-economic status. Participants from higher socio-economic status more frequently reported spontaneous recall of advertisements of three categories compared with those from low socio-economic status. While previous research has suggested that children under 18 years from lower socio-economic backgrounds are more likely to be exposed to advertisements of unhealthy foods and beverages, the evidence remains inconclusive regarding the proportion of exposure to unhealthy foods within the overall exposure^(44)^. A greater likelihood of recalling advertisements from specific product categories may be partly explained by differences in social media targeting algorithms, which can reflect variations in dietary patterns. In Uruguay, consumption of ultra-processed products and foods eaten outside the home has been found to be higher among the most affluent population segments^(45)^. Furthermore, a recent study reported a higher density of bars, restaurants and takeaway outlets in areas of medium-to-high socio-economic status compared with low socio-economic areas in Montevideo (Uruguay)^(46)^, potentially contributing to greater exposure to targeted social media posts. Considering that advertisements tailored at specific segments of adolescents may increase their persuasiveness^(47,48)^, further research is needed to explore socio-demographic differences in adolescents’ experiences with digital food marketing.

The findings of the present study stress the need to implement mandatory regulatory approaches to reduce adolescent exposure to digital marketing of unhealthy foods and beverages, as recommended by the WHO^(16,49)^. This regulatory approach has been reported to be effective in reducing purchases of foods and beverages high in sugars, saturated fat and Na^(50)^. The focus of such policies should be on exposure to any advertisement of unhealthy foods and beverages, even if it is not specifically targeted at adolescents^(51)^.

The main strength of the present study lies in its novelty, addressing an emerging topic in an underrepresented population within the food marketing literature. Methodologically, it benefits from a large sample size, a robust recruitment method and high school participation rate. However, some limitations must be acknowledged. First, the study relies on self-reported spontaneous recall, which likely captures only a portion of the digital food marketing adolescents are exposed to. Additionally, social media use was assessed through a multiple-choice question, focusing only on weekdays, which may underestimate overall media exposure, particularly on weekends when adolescents typically have more free time.

### Conclusion

The findings of the present work show that adolescents in an emerging Latin American country frequently spontaneously recall exposure to digital food marketing, particularly for unhealthy foods and beverages. This underscores the need to implement regulatory approaches to reduce adolescent exposure to digital marketing of unhealthy foods and beverages.

## Supporting information

Ares et al. supplementary material Ares et al. supplementary material

## Supplementary material

For supplementary material accompanying this paper visit https://doi.org/10.1017/S1368980025101481
