# Supplementary material for: Adolescents’ spontaneous recall of food and beverage advertisements on digital media: a cross-sectional survey in Montevideo, Uruguay
Source: Public Health Nutr. 2025 Dec 3;28(1):e203. doi: 10.1017/S1368980025101481 (PMC12780806; doi:10.1017/S1368980025101481)
Supplement: Ares et al. supplementary material [file S1368980025101481sup001.pdf]

## Supplementary material

**Table 1.** Characteristics of the adolescents who participated in the survey (n=1542).

| Characteristic | Number of participants | Percentage of participants (%) | Percentage of the participants without missing data (%) |
|---|---|---|---|
| *Gender* | | | |
| Female | 866 | 56.2 | 56.6 |
| Male | 655 | 42.5 | 42.8 |
| Other | 9 | 0.6 | 0.6 |
| Missing data | 12 | 0.8 | - |
| *Age range* | | | |
| 11-14 years old | 909 | 58.9 | 59.5 |
| 15-19 years old | 619 | 40.1 | 40.5 |
| Missing data | 14 | 0.9 | - |
| *Socio-economic status* | | | |
| Low | 214 | 13.9 | 14.1 |
| Medium | 938 | 60.8 | 61.9 |
| High | 364 | 23.6 | 24.0 |
| Missing data | 26 | 1.7 | - |
| *Digital media use* (*) | | | |
| Any digital media | 1535 | 99.5 | 99.5 |
| Website browsing | 1438 | 93.2 | 93.2 |
| YouTube | 1335 | 86.6 | 86.6 |
| Instagram | 1273 | 82.6 | 82.6 |
| TikTok | 1226 | 79.5 | 79.5 |

Note: Socio-economic status was estimated based on the score of the neighborhood of residence in the national socio-economic status index. (*) Digital media use was assessed using a check-all-that-apply question, making it impossible to evaluate missing data. Notably, all participants who reported using social media selected at least one social media platform.

**Table 2.** Description of the codes included in the coding frame developed through inductive coding to analyze responses to the open-ended question assessing spontaneous recall of food and beverage advertisements in social media.

| Category | Description |
|---|---|
| *Outlets selling prepared foods* | References to advertisements of outlets selling prepared foods, food delivery apps or specific types of fast food |
| Fast-food and fast-food restaurants | References to fast-food (e.g., burgers, French fries) or specific fast-food restaurants (e.g., MacDonalds, Burger King) |
| Food delivery apps | References to food delivery apps (e.g., *Pedidos Ya*) |
| Restaurants (generic) | Generic references to restaurants, without any mention to specific foods or beverages |
| Sushi restaurants | References to restaurants offering sushi |
| Vegan foods and restaurants | Generic references to vegan or vegetarian foods, or to restaurants that offer these options |
| *Ultra-processed products* | References to advertisements of specific ultra-processed products or brands of such products |
| Soft drinks | References to soft drinks or to brands of such products |
| Savoury snacks | References to savoury snacks (e.g., corn snacks, potato chips) or to brands of such products |
| Energy drinks | References to energy drinks or to brands of such products |
| Flavoured water | References to flavoured water or to brands of such products |
| Chocolates and confectionary | References to chocolates and confectionary (e.g., candies, chewing gum) or to brands of such products |
| Condiments | References to condiments (e.g., ketchup, mayonnaise) or to brands of such products |
| Cookies and crackers | References to cookies and crackers or to brands of such products |
| Ice-creams | References to ice-creams or to brands of such products |
| Alfajores | References to alfajores (a traditional product composed of two cookies with a filling in the middle) or to brands of such products |
| Cold cuts and sausages | References to cold cuts and sausages (e.g., ham, hot dogs) or to brands of such products |
| Bakery products | References to bakery products (e.g., bread) or to brands of such products |
| Powdered drinks and bottled juices | References to powdered drinks and bottled juices or to brands of such products |
| Dairy products | References to dairy products (e.g., yogurt, milk desserts) or to specific brands of such products |
| Instant soups and bouillon cubes | References to instant soups and bouillon cubes or to specific brands of such products |
| Breakfast cereals and cereal bars | References to breakfast cereals and cereal bars or to specific brands of such products |
| Marmalade and dulce de leche | References to marmalade or *dulce de leche*, a traditional type of sweetened condensed milk |
| *Natural foods and culinary preparations* | References to advertisements of natural foods or culinary preparations, regarded as healthy foods according to the Uruguayan dietary guidelines |
| Recipes | Generic references to recipes or typical culinary preparations |

| | |
|---|---|
| Healthy foods | Generic references to healthy foods, without any mention to specific foods |
| Fruits and vegetables | References to the generic terms ‘fruit’ and ‘vegetables’ or to specific fruits and vegetables |
| *Others* | References to other advertisements not included in the other categories |
| Desserts | Refences to specific desserts or to the general term desserts |
| Alcoholic beverages | References to specific alcoholic beverages or to the general terms ‘alcohol’ or ‘alcoholic beverages’ |
| Promotions (e.g., discounts) | References to food promotions, such as discounts and merchandising, regardless of the specific product being promoted |
| Coffee | Generic references to coffee or coffee brands |
| Weight loss | References to diets, weight loss supplements, diets, weight loss treatments, or weight loss clinics |
| Yerba mate | Generic references to *yerba mate*, a herb used to prepare a traditional infusion, or brands of *yerba mate*. |
